# Arbuscular mycorrhizal symbiosis elicits shoot proteome changes that are modified during cadmium stress alleviation in *Medicago truncatula*

**DOI:** 10.1186/1471-2229-11-75

**Published:** 2011-05-05

**Authors:** Achref Aloui, Ghislaine Recorbet, Franck Robert, Benoît Schoefs, Martine Bertrand, Céline Henry, Vivienne Gianinazzi-Pearson, Eliane Dumas-Gaudot, Samira Aschi-Smiti

**Affiliations:** 1 UMR INRA 1088/CNRS 5184/UB. Plante-Microbe-Environnement. INRA-CMSE. BP 86510. 21065 Dijon Cedex, France; 2 Département des Sciences Biologiques, Faculté des Sciences de Tunis, Campus universitaire, 1060 Tunis, Tunisia; 3 Microorganismes, Metaux et Toxicité, Institut National des Sciences et Techniques de la Mer, Conservatoire National des Arts et Métiers, BP 324, 50103 Cherbourg-Octeville Cedex, France; 4 Unité de Biochimie Bactérienne, PAPPSO, batiment 526, Domaine de Vilvert 78352, Jouy en Josas Cedex, France

## Abstract

**Background:**

Arbuscular mycorrhizal (AM) fungi, which engage a mutualistic symbiosis with the roots of most plant species, have received much attention for their ability to alleviate heavy metal stress in plants, including cadmium (Cd). While the molecular bases of Cd tolerance displayed by mycorrhizal plants have been extensively analysed in roots, very little is known regarding the mechanisms by which legume aboveground organs can escape metal toxicity upon AM symbiosis. As a model system to address this question, we used *Glomus irregulare*-colonised *Medicago truncatula *plants, which were previously shown to accumulate and tolerate heavy metal in their shoots when grown in a substrate spiked with 2 mg Cd kg^-1^.

**Results:**

The measurement of three indicators for metal phytoextraction showed that shoots of mycorrhizal *M. truncatula *plants have a capacity for extracting Cd that is not related to an increase in root-to-shoot translocation rate, but to a high level of allocation plasticity. When analysing the photosynthetic performance in metal-treated mycorrhizal plants relative to those only Cd-supplied, it turned out that the presence of *G. irregulare *partially alleviated the negative effects of Cd on photosynthesis. To test the mechanisms by which shoots of Cd-treated mycorrhizal plants avoid metal toxicity, we performed a 2-DE/MALDI/TOF-based comparative proteomic analysis of the *M. truncatula *shoot responses upon mycorrhization and Cd exposure. Whereas the metal-responsive shoot proteins currently identified in non-mycorrhizal *M. truncatula *indicated that Cd impaired CO_2 _assimilation, the mycorrhiza-responsive shoot proteome was characterised by an increase in photosynthesis-related proteins coupled to a reduction in glugoneogenesis/glycolysis and antioxidant processes. By contrast, Cd was found to trigger the opposite response coupled the up-accumulation of molecular chaperones in shoot of mycorrhizal plants relative to those metal-free.

**Conclusion:**

Besides drawing a first picture of shoot proteome modifications upon AM symbiosis and/or heavy metal stress in legume plants, the current work argues for allocation plasticity as the main driving force for Cd extraction in aboveground tissues of *M. truncatula *upon mycorrhization. Additionally, according to the retrieved proteomic data, we propose that shoots of mycorrhizal legume plants escape Cd toxicity through a metabolic shift implying the glycolysis-mediated mobilization of defence mechanisms at the expense of the photosynthesis-dependent symbiotic sucrose sink.

## Background

Cadmium (Cd) is a widespread hazardous heavy metal, whose release in the plant environment has been dramatically accelerated by anthropogenic activities such as mining, refining, and soil amendments with sewage sludge and phosphate fertilizers [1]. Cd is generally harmful to most plant species in which its accumulation induces leaf chlorosis, root necrosis and decreases in growth and tissue-size [2]. The main known mechanisms of Cd ion (Cd^2+^) toxicity in living organisms include its affinity for sulfhydryl groups in proteins and its ability to replace some essential metals in active sites of enzymes, thus causing inhibition of enzyme activities and protein denaturation [2,3]. Oxidative stress also belongs to Cd-induced plant cell responses as a consequence of interference with antioxidant enzymes and depletion of antioxidant molecules [4], which may result in oxidative damages to phospholipid membranes, proteins and DNA [5,6]. Several mechanisms susceptible to counteract Cd toxicity have been identified in plants including active efflux and reduced transport at the plasmalemma, metal chelation by high-affinity ligands such as phytochelatins, glutathione and metallothioneins, and compartmentalization into the vacuole [7]. Besides these intracellular processes, exudates secretion, metal binding to the cell wall and rhizospheric microorganisms also have the potential to contribute to plant defence mechanisms against Cd toxicity [2,8,9].

Notably, arbuscular mycorrhizal (AM) fungi, which engage a mutualistic symbiosis with the roots of most plant species, *Arabidopsis *(Brassicaceae) belonging to the noticeable exceptions, have received much attention for their ability to increase heavy metal tolerance in plants [2,10,11]. By enlarging the volume of soil explored by the roots thanks to an extensive extra-radical network, these ubiquitous soil borne microorganisms can increase plant phosphate, micronutrient and water uptake [12]. In turn, AM fungi that are obligate plant biotrophic microorganisms are supplied with the organic carbon forms essential for them to achieve their full life cycle [13]. Actually, a substantial amount of photosynthetically fixed carbon is channelled for synthesis of sucrose, which after cleavage represents the main source of hexoses translocated to AM fungi [14]. As a result, the sucrose symbiotic sink diverts the flow of triose phosphates produced through the Calvin cycle to feed mycorrhizal intraradical structures. Depending on the combination of host, fungus and heavy metal, phytostabilization and/or phytoextraction can contribute to alleviate metallic stress in mycorrhizal plants: in the former case, heavy metals are immobilized in the rhizosphere through precipitation in the soil matrix, adsorption onto the root surface or accumulation within roots, whereas in the latter, heavy metals are compartmentalized in plant aboveground parts through root-to-shoot transfer mechanisms and/or increased biomass production [11].

Nonetheless, researches regarding shoot tolerance mechanisms upon heavy metal phytoextraction have been essentially conducted in hyperaccumulator plant species; so that there is little evidence regarding those processes by which mycorrhiza allow plant shoots to cope with metal stress [15]. Actually, probably because roots are considered as the main site of metal toxicity exposure, the cellular and molecular bases of Cd tolerance of mycorrhizal plants have been essentially grasped at the belowground level [16-22]. Although it has been demonstrated that mycorrhizal legumes can accumulate and tolerate Cd in their aboveground organs [11,22,23], and despite evidences for a role of plant aerial organ proteins in heavy metal stress tolerance (reviewed in [24]), large-scale data are lacking regarding the molecular mechanisms by which shoots of mycorrhizal legumes can escape Cd toxicity. In a previous study, we reported on the protective effect conferred by AM symbiosis to *M. truncatula *plants grown in Cd-contaminated substrate with regard to plant biomass and phytotoxicity symptoms [22]. Because mycorrhizal *M. truncatula *plants also were found to accumulate Cd in their shoots, we choose this system as a model to investigate the mechanisms by which legume shoots tolerate Cd toxicity upon AM symbiosis. In the present work, to complement the physiological parameters that were measured to obtain structural and functional information on the impact of Cd and/or mycorrhization on plant biomass and photosynthetic activity, a two-dimensional electrophoresis (2-DE)-based study was further used to compare *M. truncatula *shoot responsive proteins upon AM colonization and Cd application. We report on a first picture of plant legume shoot proteome modifications displayed upon AM symbiosis and their modulation in response to Cd stress, which allowed proposing a working model to explain heavy metal tolerance in aboveground organs of legume mycorrhizal plants.

## Results and Discussion

### Shoots of mycorrhizal *M. truncatula *plants have a capacity for extracting Cd, which is not related to an increase in root-to-shoot translocation rate but to allocation plasticity

**Figure 1:**
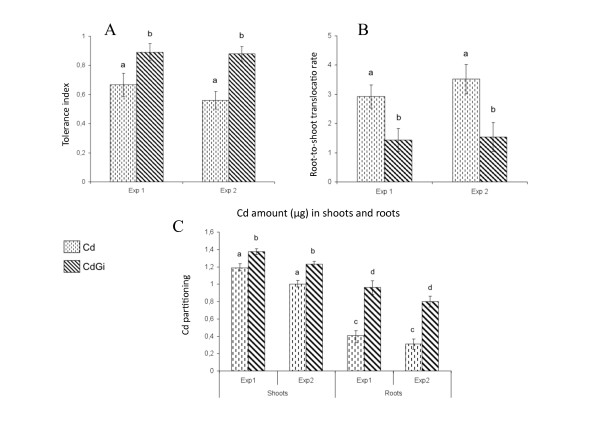
**Effectiveness in cadmium phytoextraction of 3-wk old *M. truncatula *plants colonised by *G. irregulare *(CdGi) relative to those non-inoculated (Cd), as measured by shoot tolerance index (A), root-to-shoot transport factor (B), and metal partitioning in shoot and root tissues (C)**. The tolerance indices for shoots of mycorrhizal and non-mycorrhizal *M. truncatula *plants were calculated as the ratio of biomass (g fresh weight) for plants grown in Cd-spiked substrate to plants grown in Cd-free substrate. The contribution of shoots to plant biomass was measured as the ratio of shoot biomass (g fresh weight) to that of whole plant. Root-to-shoot translocation rates for Cd were calculated in mycorrhizal and non-mycorrhizal plants as the ratio of the Cd amount (μg) in shoots to that in root. Cd partitioning indices corresponded to the metal quantities (μg) mobilized in plant organs. For two independent biological experiments (Exp), histograms represent means of three replicates (means ± SD, n = 3). Means marked with different letters indicate significant difference at *p *< 0.05.

**Figure 2:**
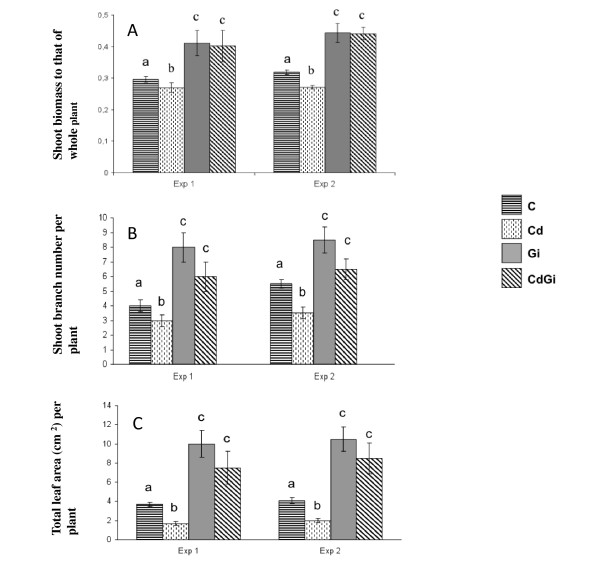
**Impact of cadmium (Cd) and/or *G. irregulare *inoculation (Gi) on shoot biomass allocation patterns of 3-wk old *M. truncatula *plants relative to those non-treated (C), as measured by the ratio of shoot biomass (g fresh weight) to that of whole plant (A), shoot branch number per plant (B), and total leaf area (cm^2^) per plant (C)**. For two independent biological experiments (Exp), histograms represent means of three replicates (means ± SD, n = 3). Means marked with different letters indicate significant difference at *p *< 0.05.

**Figure 3:**
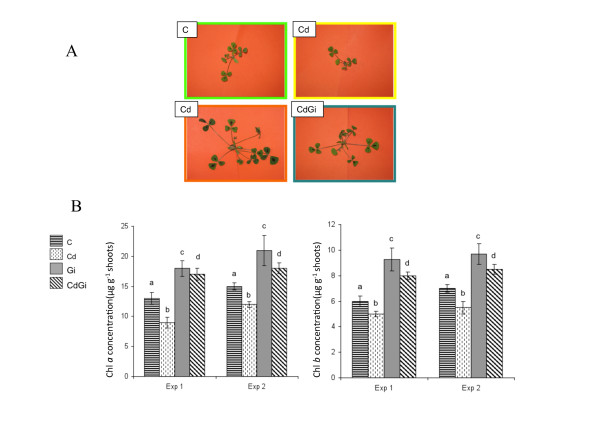
**Impact of cadmium (Cd) and/or *G. irregulare *inoculation (Gi) on leaf aspect (A) and chlorophyll (Chl) *a *and *b *concentrations (μg.g^-1 ^of shoots) (B) of 3-wk old *M. truncatula *plants relative to those non-treated (C)**. For two independent biological experiments (Exp), histograms represent means of three replicates (means ± SD, n = 3). Means marked with different letters indicate significant difference at *p *< 0.05.

To go further in analysing the tolerance to Cd displayed by *M. truncatula *when colonised by the AM fungus *Glomus irregulare *[22], we have investigated in the current study the potential contribution of mycorrhizal plants to phytoextraction, which refers to the transfer of inorganic contaminants from soil to harvestable aboveground plant tissues [25]. Because phytoextraction relies on the capacity of plants to accumulate and tolerate heavy metals in their shoots, its efficiency depends both on the ability of metal to be translocated in aerial plant organs and on shoot biomass production [25-27]. To take into account biomass production, which actually escapes indices calculated on the basis of Cd concentration [27,28], three main indicators have been used to measure plant effectiveness in extracting Cd from soil: the tolerance index expressed as the ratio of shoot growth parameters for plants grown in polluted soil to plants grown in metal-free soil [26,27,29], the transport factor calculated as the ratio of the total Cd amount in shoots to that in roots [27,30], and Cd partitioning that corresponds to the metal quantity present in plant organs [28]. When calculated for *M. truncatula *plants grown for three weeks in Cd-spiked substrate (2 mg kg^-1^) in the presence or not of *G. irregulare*, the first indicator showed that mycorrhizal plants displayed a reproducible and significant increase in shoot tolerance to Cd relative to those non-mycorrhizal (Figure 1A), a result corroborating a previously reported enhanced *M. truncatula *aboveground biomass in Cd-contaminated soils when inoculated with *G. irregulare *[28]. Concomitantly, although the root-to-shoot translocation rate of Cd was significantly reduced by two-fold in *G. irregulare*-colonised plants compared to those non-inoculated (Figure 1B), reflecting the important role of mycorrhizal roots in heavy metal sequestration [22], Cd partition patterns (Figure 1C) indicated that shoots of mycorrhizal plants contained the highest metal quantity relative to shoots of non mycorrhizal plants and *M. truncatula *roots, either *G. irregulare*-colonised or not. From these three indicators for metal phytoextraction, it could be concluded that shoots of mycorrhizal *M. truncatula *plants have a capacity for extracting Cd, which is not related to an increase in root-to-shoot transport factor, a result previously observed in Cd-treated mycorrhizal sunflowers plants [31]. As a strategy for metal phytoextraction alternative to increase in root-to-shoot transfer mechanisms, it has been proposed that a significant shift in root-to-shoot biomass partitioning permitted some plants to reduce the incidence of metal-induced stress in photosynthetic organs, a process referred to as allocation plasticity [32]. At the same time, an increased tolerance to metal of the photosynthetic system would allow plants maintaining high transpiration efficiency, thus creating a water flux that can drive metals from the roots into the stem and leaves where the metal can be compartmentalized [33]. To test whether this model appropriated to Cd-treated mycorrhizal *M. truncatula *plants, we thus compared between treatments the biomass allocation pattern and the chlorophyll (Chl) content as an indicator of metal-induced damage to the photosynthetic apparatus. With regard to root-to-shoot partition in *M. truncatula *plants upon Cd exposure and/or mycorrhizal infection (Figure 2A, Additional file 1), it happened that whereas Cd application reduced the biomass allocated to the shoots in non-mycorrhizal plants relative to control, mycorrhization led to its significant increase both in the absence and presence of metal. Further measurements indicated that Cd-induced decrease in root-to-shoot biomass allocation in non-mycorrhizal plants accounted for a significant reduction both in shoot branching and leaf area per plant (Figure 2B-C). On the contrary, *G. irregulare *induced relative to non-mycorrhizal plants significant increases in shoot branching and leaf area, which were not significantly affected by Cd treatment (Figure 2B-C). In *Trifolium repens*, a higher leaf area in response to mycorrhization has been shown to maximize the area available for CO_2 _assimilation per unit of carbon invested, and thus the rate of photosynthesis [34]. In the current study, although none of the Cd-treated plants, either mycorrhizal or not, exhibited a yellowing symptom indicative of leaf chlorosis usually associated with Cd induced-damage (Figure 3A), significant differences in Chl content between treatments could be reproducibly recorded in plant shoots. As shown in Figure 3B, Cd led to a significant reduction in the contents of Chl *a *and *b *in non-mycorrhizal plants, which has been ascribed for example to the inhibition of aminolevulinic acid synthesis and protochlorophyllide photoreduction [35,36]. By contrast, a roughly two-fold increase in Chl contents was measured upon AM colonisation of the root system relative to control plants, which was not significantly affected in the presence of Cd.

**Figure 4:**
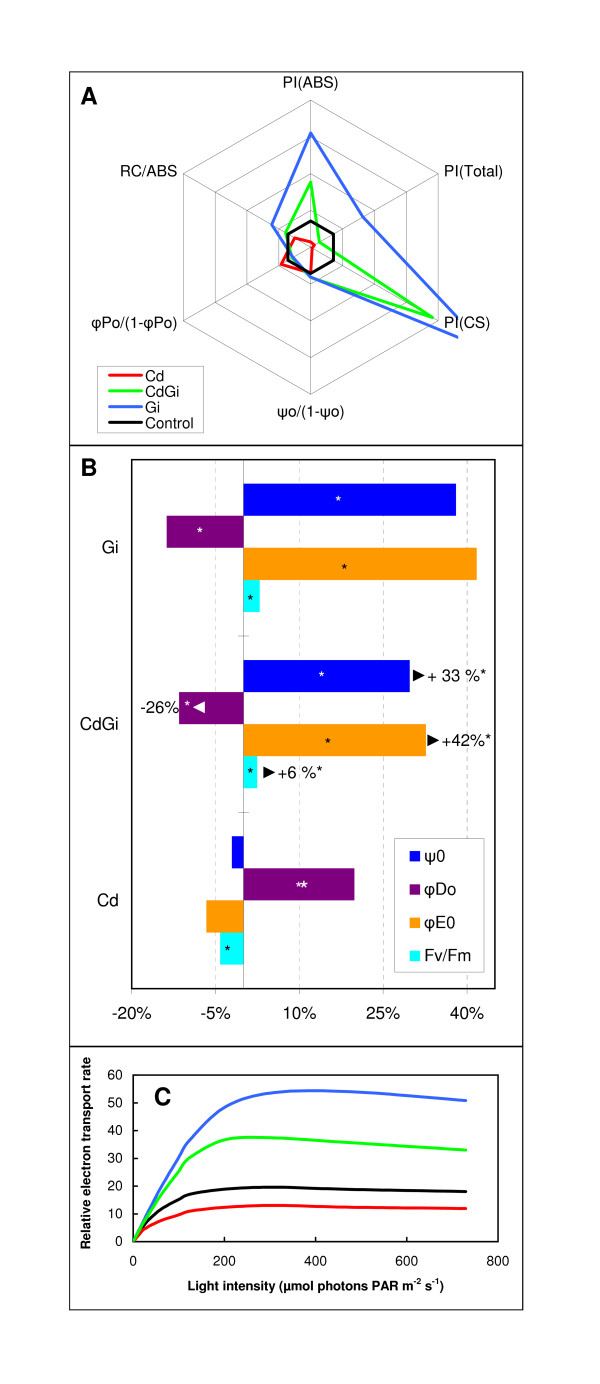
**Influence of cadmium (Cd) and/or *G. irregulare *inoculation (Gi) on the parameters of the OJIP-test and relative electron transport rate**. **(A)**, Variations of performance indices and structural OJIP-test parameters plotted (spiderplot center = 0.5, maximum = 4) relative to their respective control (set as reference ticked line = 1). **(B)**, Deviation of the fluxes as expressed relative to non-treated plants. Asterisks indicate significant difference at *p *< 0.05. The values indicated by arrows, expressed in percents, reflect the deviation flux differences between Cd-treated mycorrhizal plants and those only Cd-treated. Asterisks indicate significant difference at *p *< 0.05. Data relative to panels A and B represent an average of 5 to 6 independent measurements. The corresponding statistical data (n ≥ 5) are provided in additional file 2. **(C)**, Plot of relative of relative linear electron transport rate (ETR) as a function of quantum flux density of the photosynthetically active radiation (PAR). The ETR was measured via the fluorescence parameter (as described in Methods) in attached leaves. Data represent an average of six plant replicates. The colour legend of panel C is the same as for panel A. Statistical data (n = 6) are provided in additional file 2 for the maximum apparent ETR (ETR_MAX_).

To determine whether the modifications in the Chl amount reported in Figure 3 impacted the photosynthetic activity, photosynthetic efficiencies, vitality indices, and the relative electron transport rate (ETR) were measured [37,38]. As presented in Figure 4 and additional file 2 for related statistics, the performance index for energy conservation from photons absorbed by PSII to the reduction of PSI end acceptors (PI_TOT_), and for energy conservation from photons absorbed by PSII to the reduction of intersystem electron acceptors, expressed per reaction centre (PI_ABS_) or per leaf cross section (PI_CS_) were decreased by approximately 50% in response to Cd in non mycorrhizal plants (Figure 4A). A decrease of PI_ABS _was also reported to occur in pea leaves upon Cd-treatment [26]. After calculation of the energy flux ratios, panel B of Figure 4 showed that the presence of Cd in non-mycorrhizal plants reduced significantly the maximum quantum yield of photosystem 2 (F_V_/F_M _or φ_Po_) relative to controls. Accordingly, quantum yield for electron transport (φ_Eo_) and the probability that an electron moves further than Q_A_^- ^(ψ_0_) were reduced in metal-treated non-mycorrhizal plants when compared to control samples (Figure 4D). Consequently, the maximum quantum yield of the non-photochemical deexcitation (φ_Do_) increased significantly in the presence of Cd (Figure 4B). These results are in line with those obtained with other photosynthetic organisms and higher Cd concentrations [39]. Additionally, Figure 4C also showed that both the rate and the saturation of the light photosynthetic performance were lower in Cd-treated plants compared to controls, thus indicating that the capacity of the antenna to harvest photons is influenced by the Cd-induced modifications in the Chl content (Figure 3). Altogether these data show that the reason for the reduced growth of Cd-treated non-mycorrhizal plants resides partly in an altered functioning of photosynthesis but also in the lowering of the density of RC. Contrasting with the effects of Cd, the presence of *G. irregulare *in metal-free plants relative to controls promotes the development of the photosynthetic apparatus as shown by the increase in the vitality indices PI_ABS_, PI_CS _(Figure 4A) and relative ETR (Figure 4C). The PI_ABS _and PI_CS _increases are due to the significant augmentation of the density of RCs per Chl (RC/ABS) whereas the other factors composing this vitality index were quantitatively less impacted by the presence of the mycorrhizal fungus (Figure 4A). The photosynthetic capacity of mycorrhizal plants also is enhanced relative to controls as shown by the significant higher maximum quantum yield of photosystem 2 (F_V_/F_M_), the probability that an electron moves further than Q_A_^- ^(ψ_0_) and the quantum yield for electron transport (φ_Eo_), and the lower level of the quantum yield of the nonphotochemical deexcitation (φ_Do_) (Figure 4B). Overall, the results show that the presence of the arbuscular fungus promotes photosynthesis by increasing the plant ability to use light energy, facilitating the electron transport and by increasing the density of photosynthetic units. Noteworthy, when analysing the photosynthetic performance in metal-treated mycorrhizal plants relative to those only Cd-supplied (CdGi *versus *Cd), it turned out that the presence of *G. irregulare *partially alleviated the negative effects of Cd on photosynthesis. Actually, the vitality indices PI_ABS _and PI_CS _(Figure 4A) and relative ETR (Figure 4C) were significantly higher in Cd-treated mycorrhizal plants relative to those only Cd-supplied. An enhanced photosynthetic capacity of Cd-treated mycorrhizal plants compared to those only Cd-amended also is mirrored in panel B of Figure 4 by a significant higher value for parameters (F_V_/F_M_), (ψ0) and (φ_EO_) and a significant lower level for (φ_Do_).

**Figure 5:**
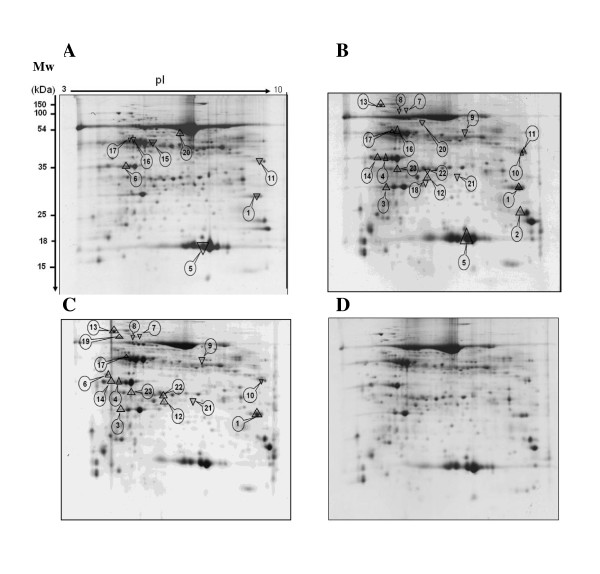
**Impact of cadmium (Cd) and/or *G. irregulare *inoculation (Gi) on 2-DE shoot proteins profiles of *M. truncatula *relative to those non-treated**; 600 μg phenol-extracted proteins were separated onto first-dimensional pH 3-10 non-linear IPG strips and second dimensional 12% vertical slab gels. Mw, Bio-Rad 2-DE molecular mass standards. Representative gels from Cd-treated plants (**A**), *G. irregulare*-inoculated plants (**B**), *G. intraradices*-inoculated Cd-treated plants (**C**) and non-treated control plants (**D**). Up- and down-headed arrows represent significant (n = 3, *p *< 0.05) increased and decreased protein abundance reproducibly quantified in two independent biological experiments compared to non-treated control plants, respectively.

**Figure 6:**
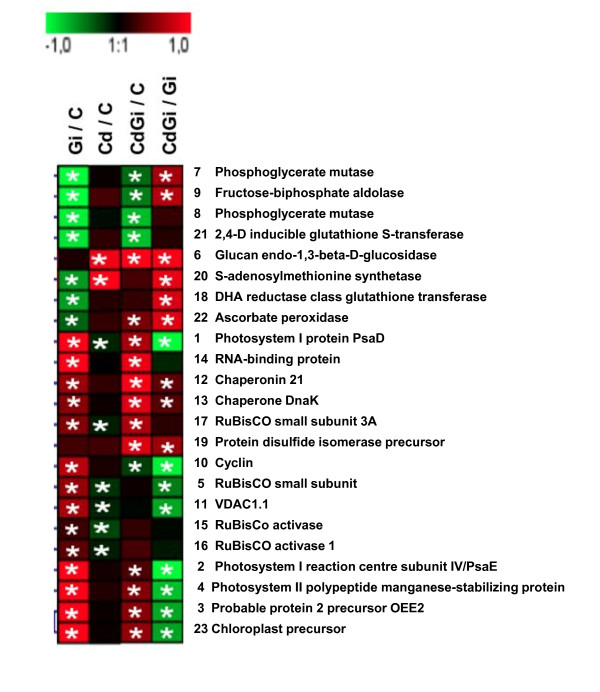
**Comparison of the groups of protein spots that responded to *G. irregulare *inoculation and/or cadmium treatment in 3-w old *M. truncatula *shoots**. Clustered abundance pattern using GENESIS software of the 23 shoot proteins of *M. truncatula *reproducibly detected as differentially accumulated (n = 3) in two independent biological experiments in response to one or both treatments. Log2 expression ratios were calculated for the different treatments (Cd, Gi and CdGi) either relative to the control reference (C) or to *G. irregulare*-inoculated plants (Gi). The green colour (-1) indicates the highest down-accumulation and the red colour (+1) the highest up-accumulation. Dark boxes (0) indicate no changes in protein abundance compared to control condition. Each row of coloured boxes is representative of a single protein and each ratio per treatment is represented using a single column, Gi/C: *G intraradices*-inoculated compared to control, Cd/C: Cd-treated compared to control, CdGi/C: *G irregulare*-inoculated and Cd-treated compared to control and CdGi/Gi: Cd treated and *G. irregulare*-inoculated compared to *G. irregulare-*inoculated plants. Asterisks indicate significant differences (*p *< 0.05). Protein spots are numbered according to Figure 5.

Taken together, both biomass partitioning- and photosynthesis-related indicators support the idea that mycorrhizal *M. truncatula *plants extract and tolerate Cd by displaying a high level of allocation plasticity. According to this model, it is also assumed that metal-treated mycorrhizotrophic plants don't invest in an intrinsic tolerance mechanism typical of *A. thaliana*, which involves for example phytochelatin production [29]. To investigate this hypothesis, we have performed a 2-DE-based proteomic comparative analysis of the *M. truncatula *shoot responses upon mycorrhization and Cd exposure, a strategy that was proved successful to unravel and discriminate the mechanisms of metal toxicity/tolerance in different plant species and/or organs [24]. Following separation of phenol-extracted shoot proteins on 2-DE gels and Coomassie Brilliant Blue-staining, protein patterns were analysed using Progenesis workstation, resulting in the detection of 500 distinct spots displayed on a virtual reference 2-DE map (data not shown). After using SameSpots and *post-hoc *statistical tests, 46 proteins, displayed on Figure 5, showed a significant (*p *< 0.05) change in abundance in treated plants relative to controls, which were reproducible in the two independent biological experiments performed in the current study, each encompassing three replicates of the four treatments. Comparison of shoot responsive proteins between the three treatments revealed that the 46 significant modifications encompassed 23 distinct spots by reason of overlapping changes (Figure 6). Subsequent MALDI-TOF analyses allowed retrieving confident positive hits for all the 23 proteins of interest, and, with the exception of spot 23, all had known or homology-inferred functions (Additional files 3 and 4). When comparing, on the basis of GENESIS clustering, the groups of proteins that responded to Cd treatment (Cd), *G. irregulare *inoculation (Gi), and Cd treatment in mycorrhizal plants (CdGi) relative to non-treated plants (C), Figure 6 showed that *G. irregulare *elicited shoot protein modifications opposite to those induced by Cd in non-mycorrhizal plants, but also that the *G. irregulare*-responsive shoot proteome was qualitatively conserved but quantitatively modified upon Cd exposure (Figure 6).

### *G. irregulare *elicits shoot proteome changes opposite to those induced by Cd

Although transcript profiling performed in plant shoots has previously indicated the systemic induction of many plant genes upon mycorrhization [40], the current study allowed demonstrating that a mycorrhiza-responsive shoot proteome also exists in *M. truncatula*. Namely, in the absence of Cd, 21 proteins were reproducibly detected as being significantly differentially accumulated in the shoots of mycorrhizal plants relative to those grown in the absence of *G. irregulare *(Figure 5B, Figure 6: column Gi/C). Among those displaying a higher abundance upon mycorrhization, were proteins having role in assimilation of CO_2 _(s5, s17, s15, s16), or belonging to photosystem I (s1, s2), and photosystem II (s3, s4), which sustains the view [34] of an increased photosynthetic activity in response to AM fungi. As obligate biotrophs, AM fungi receive their entire carbon requirements from a 4 to 30% proportion of plant photoassimilates [41], and consequently, photosynthetic activity is optimised for symbiosis demand [42]. This is mirrored by the increased abundance in mycorrhizal plants of proteins having role in cellular division (cyclin (s10)) and protein synthesis (RNA-binding precuror (s14)) that can help in sustaining shoot and leaf growth. Conversely, chaperonins 21 and DnaK (s12, s13), which protect newly synthesized or stress-denatured polypeptides from misfolding and aggregation [43], were up accumulated in response to symbiosis. We also noticed a reduced abundance in shoots of mycorrhizal plants of the gluconeogenesis/glycolysis-related proteins phosphoglycerate mutases (s7, s8) and fructose-biphosphate aldolase (s9), which operate downstream of phosphofructokinase during glycolysis (Figure 5B, Figure 6). These results corroborate the known regulation mechanism of the Calvin cycle, which is prevented from functioning in a futile reaction through a reduction in glucose breakdown *via *a decreased glycolysis, a process that is achieved by the light-driven electron flow production of reduced thioredoxin, which inhibits the glycolytic enzyme phosphofructokinase [44,45]. Upon mycorrhization, we additionally observed in *M. truncatula *shoots the up-accumulation of a mitochondrial voltage-dependent anion channel (VDAC, s11), for which reduction in abundance is regarded as a marker of oxidative damage [46,47], coupled to the down-accumulation of four proteins having role in counteracting oxidative stress (2,4D inducible glutathione transferase (s21), S-adenosylmethionine synthetase (s20), DHA reductase class glutathione transferase (s18), ascorbate peroxidase (s22)), which indicated a decreased need in mechanisms scavenging reactive oxygen species (ROS) in mycorrhizal plants. Actually, non-enzymatic antioxidants include the major cellular redox buffers ascorbate and glutathione (GSH). Whereas GSH is oxidized by ROS to form glutathione disulfide (GSSH), ascorbate is oxidized via ascorbate peroxidase (APX) to form monodehydroascorbate (MDA) that dismutates into dehydroascorbate (DHA). Through the ascorbate-glutathione cycle, DHA can be reduced by DHA reductase reforming GSH and ascorbate [48]. Glutathione S-transferases (GSTs) also participate in the detoxification of reactive electrophillic compounds by catalysing their conjugation to GSH. Among GSTs, the DHA reductase class has a specialized function in reducing dehydroascorbate to ascorbic acid [49]. Finally, S-adenosylmethionine synthetase is an enzyme catalysing the formation of S-adenosylmethionine (SAM) from methionine and ATP. SAM serves as a precursor of nicotianamine, for which a role in metal ion homeostasis through chelation mechanisms has been reported [50,51], and as a key substrate of certain methylases for the regeneration of GSH [52,53]. Notably, among the aforementioned proteins, both APX and DHA reductase are known participants of the water-water cycle, also referred to as the Mehler reaction or Asada pathway [54], which plays a critical role in protecting the photosynthetic apparatus from photo-oxidative damage by dissipating excess light energy off the electron transport chain through the ascorbate peroxidase pathway. Besides APX and DHA reductase, ascorbate, glutathione and GSTs also have function in counteracting photoinhibition in the presence of excess excitation energy [54]. According to the down-accumulation of APX, DHA reductase, GST and SAM synthetase in plant shoots upon *G. irregulare *inoculation, we propose that the ascorbate-gluthatione cycle is less operative in mycorrhizal plants and that mycorrhization helps in reducing oxidative stress in photosynthetic organs. In favour of this hypothesis were the mycorrhization-induced increases in CO_2 _assimilation (s15, s16, s17), protein-pigment assembly (s1, s2, s3, s4, s5), which contribute in dissipating absorbed photon flux through the sufficient production of reducing equivalents [55]. Concomitantly, a significant decrease in the maximum quantum yields of nonphotochemical deexcitation and increase in the relative ETR were observed upon mycorrhization relative to control plants (Figure 4). Collectively, these data indicate that the mycorrhiza-responsive shoot proteome of *M. truncatula *is characterised by a reduction in antioxidant-related mechanisms and by an increase in photosynthesis-related processes that drive a decreased glycolytic flux.

Despite the model status of *M. truncatula *for legumes, untargeted approaches aiming at deciphering its molecular responses upon heavy metal exposure remain scarce [22] relative to those performed with its non-mycorrhizotroph counterpart *Arabidopsis *(reviewed in [24]). In the current work, by contrast to what observed upon mycorrhization in *M. truncatula *shoots (Figure 5A, Figure 6, column Gi/C), Cd triggered the down- and up-accumulation of VDAC1 (s11) and SAM synthetase (s20), respectively (Figure 6, column Cd/C), a pattern that was ascribed to a plant defence response against Cd-induced oxidative stress. In shoots of non-mycorrhizal plants, an increased accumulation of the pathogenesis-related (PR) protein β-glucanase (s6) was also observed upon Cd exposure (Figure 6, column Cd/C). Metal ions are not only well known to induce defence-related proteins as a result of cell-damaging actions, but also they share common signalling molecules with biotic stresses such as ethylene, salicylic acid and jasmonic acid [56]. Even though Cd is not a redox-active metal *per se *[1], Cd can mediate the formation of ROS including superoxide anion and hydrogen peroxide by disrupting the balance between ROS generation and the antioxidant system activity [6,57]. In plants, Cd-induced impairment of photosynthesis is considered as a one of the main causes of ROS production [58-60]. In *M. truncatula*, not only Cd reduced the Chl content in shoots of non-mycorrhizal plants (Figure 3), but also negatively affected both light-independent and dependent reactions of photosynthesis, as inferred from the decreased abundance of RuBisCO subunits, RuBisCO activases, and protein PsaD (Figure 5A, Figure 6: column Cd/C). Despite the major role attributed to the ascorbate-glutathione cycle in ROS alleviation, none of the proteins related to this process happened to belong to the Cd-responsive shoot proteome of *M. truncatula *(Figure 6, column Cd/C), indicating that this pathway might not be operative upon Cd exposure in our experimental conditions. In favour of this hypothesis was the down-accumulation of PsaD that was observed in the shoots of Cd-treated *M. truncatula *plants (Figure 6, column Cd/C). PsaD is a small extrinsic polypeptide of the PSI reaction centre complex that is required for native assembly of PSI reaction clusters [61]. A reduced electron flux due to PSI disassembly can generate direct photoreduction of oxygen, which leads to inactivation of ROS-scavenging enzymes and CO_2 _fixation [54,61,62]. PSI disassembly could further drive the destruction of PSII unit and thus a reduction in the density of active RC as presented in Figure 4. Overall, the metal-responsive shoot proteins currently identified in *M. truncatula *supports the view that an environmentally relevant supply of Cd impairs photosynthesis and generates an oxidative stress that cannot be efficiently counteracted (Figure 6, column Cd/C).

### The *G. irregulare*-responsive shoot proteome is qualitatively conserved but quantitatively modified upon Cd exposure

Following the analysis of the *M. truncatula *shoot proteome displayed in response to mycorrhization and Cd treatment (Figure 5C), 17 proteins showed a significant (*p *< 0.05) change in abundance relative to control plants, which was reproducible in the two biological experiments that were performed (Figure 6, column CdGi/C). Encompassing up-accumulation of proteins having function in the photosynthetic assimilation of CO_2_/electron transport/structural proteins (s17, s1, s2, s3, s4), together with down-accumulation of glugoneogenesis/glycolysis-related enzymes (s7, s8, s9), the recorded modifications sustain, as discussed above, an increase photosynthetic activity (Figure 4) and the concomitant silencing of the glycolytic pathway in response to mycorrhization and Cd exposure. At the same time, cell defence-related mechanisms also were raised up, as mirrored by the increased abundance of PR protein (s6), APX (s22) and protein disulfide isomerase (PDI, s19), the latter facilitating protein folding via disulfide bond isomerization during *de novo *protein synthesis and the reassembly of molecules denatured by stress [63]. From the comparison of the *M. truncatula *shoot proteins that responded to the three different treatments (Figure 6, columns Cd/C, Gi/C, CdGi/C), the shoot proteome of Cd-treated mycorrhizal plants (CdGi/C) turned out to qualitatively resemble more that observed upon mycorrhization (Gi/C) than upon Cd supply (Cd/C) alone (Figure 6). Notably, out of the 17 proteins that changed in abundance in response to mycorrhization and Cd exposure (column CdGi/C), 13 spots (s7, s9, s8, s21, s1, s14, s12, s13, s17, s2, s4, s3, s23) displayed an accumulation pattern overlapping with mycorrhiza-related proteins (column Gi/C), whereas only one protein (PR protein s6) was reminiscent of the Cd-responsive shoot proteome (column Cd/C). Taken together, these observations substantiate the idea that the mycorrhiza-responsive proteome dominates that elicited by Cd, as previously observed in metal-treated mycorrhizal roots [22].

**Figure 7:**
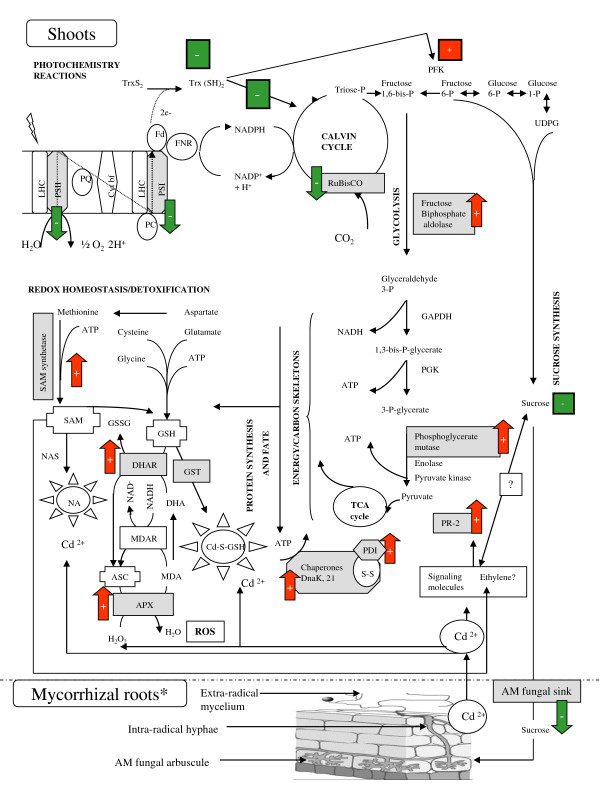
**Schematic representation of the model proposed to explain cadmium stress alleviation in shoots of *M. truncatula *plants upon AM symbiosis**. on the basis of the proteins and parameters identified in the current study as differentially accumulated in response to *G. irregulare *inoculation and/or metal treatment. Plant components identified in this work and explicit for this model are indicated in grey-shaded boxes. Red-shaded up- and green-shaded down-headed arrows indicate significant (n = 3, *p *< 0.05) increased and decreased component abundance in Cd-treated mycorrhizal plants relative to those only inoculated with *G. irregulare*, respectively. Green or red-shaded boxes indicate generally known mechanisms of regulation. Inferred metabolic pathways and intermediates are shown in non-shaded bold and non-bold letters, respectively. AM, arbuscular mycorrhizal; ASC, ascorbate; APX, ascorbate peroxidase; Cd^2+^, cadmium ion; DHA, dehydroascorbate; DHAR, dehydroascorbate reductase; Fd, ferredoxin; FNR, ferredoxin-NADP^+ ^reductase, GAPDH, glyceraldehyde-3-phosphate dehydrogenase; GSH, reduced glutathione; GSSH, glutathione disulfide; GST, glutathione S-transferase; LHC, light harvesting complex; MDA, monodehydroascorbate; MDAR, monodehydroascorbate reductase; NA, nicotianamine; NAS, nicotianamine synthase; P, phosphate; PC, plastocyanin; PDI, protein disulfide isomerase; PFK, phosphofructokinase; PGK, phosphoglyceraldehyde kinase; PQ, plastoquinone; PR-2, class 2 pathogenesis-related protein; PSI, photosystem I, PSII, photosystem II; ROS, reactive oxygen species; SAM, S-adenosylmethionine; TCA, tricarboxylic acid; TrsS_2_, thioredoxin disulfide; Trx(SH)_2_, reduced thioredoxin; UDPG, uridine diphosphoglucose;*, modified from [86]; ╬ indicates molecules having antioxidant properties; ☼indicates molecules having metal chelation properties.

The retrieved proteomic data also showed that part of AM symbiosis-related shoot proteins was quantitatively modified in presence of Cd, as inferred from the analysis of the Cd-responsive mycorrhizal proteome (Figure 6, column CdGi/Gi). Besides the increased abundance of β-glucanase (s6), Cd was also found to trigger in shoot of mycorrhizal plants relative to those *G. irregulare*-colonised but metal-free, the down-accumulation of cyclin (s10), VDAC1 (s11), RuBisCO small subunit (s5), PSI- and PSII-related proteins (s1, s2, s3, s4), coupled to the up-accumulation of gluconeogenesis/glycolytic enzymes (s7, s9), antioxidant-related proteins (SAM synthetase (s20), DHA reductase (s18), APX (s22)) and molecular chaperones (s12, s13, s19). From these observations, we propose that legume shoots of mycorrhizal plants escape Cd toxicity through the glycolysis-dependent mobilization of SAM synthetase, DHA reductase, APX and chaperones at the expense of the symbiotic sucrose sink. According to this model schematised in Figure 7, allocating less sucrose to the root inhabiting AM fungus would permit plants to retain more carbohydrates in their leaves to sustain repair mechanisms in response to foliar stress. Actually, when sucrose export is inhibited, sugars accumulate in the leaf [64] and down regulate photosynthesis to maintain homeostasis [48,65], a process mirrored in the current study by the decreased abundance in CO_2 _assimilation-proteins (RuBisCO small subunit (s5), PSI- and PSII-related proteins (s1, s2, s3, s4) together with the reduced vitality indices and relative electron transport rate in Cd-treated mycorrhizal plants relative to those mycorrhizal but metal-free (Figure 4 and 6:column CdGi/Gi). Likewise, plants that exhibit a strong accumulation of hexoses in source leaves have been shown to display an increase defence status in their aboveground organs concomitant to an undersupply of carbohydrates to the roots and a reduced mycorrhization [66,67]. The assumption of a decreased sink demand is supported in our experimental conditions by the 50% lower development of the mycobiont in the roots of Cd-treated *M. truncatula *[22]. Consecutive to a minimised sucrose export to root sink tissues and the subsequent down-regulation of photosynthesis, chemical energy and carbon skeletons can be freed up as a source of metabolites to be used in a defence response through the glycolytic pathway *via *the activation of the enzyme PFK and the down-regulation of the energy-consuming gluconeogenesis process [48]. Namely, concomitant to the up-accumulation of fructose biphosphate aldolase and phosphoglycerate mutase (s7, s9), was an increased abundance in SAM synthetase, DHA reductase, APX, chaperones in Cd-treated mycorrhizal plants relative to those metal-free (Figure 6, column CdGi/Gi), which have function in counteracting Cd-induced oxidative stress and/or in assisting folding during *de novo *protein synthesis. Overall, the proposed model implies a major role for the ascorbate-gluthatione cycle and molecular chaperones in Cd stress alleviation in shoots of mycorrhizal *M. truncatula *plants, but also presupposes that the mycorrhiza-induced sucrose sink and the concomitant increase in photosynthesis may be key actors in generating a carbon reserve to be mobilized in case of foliar stress (Figure 7). This idea is partly reminiscent of the "fuel for the fire" concept that illustrates plant leaf metabolism upon pathogen infection, in which carbohydrate increase is thought to be a metabolic signal that induces the expression of defence-related genes and repression of photosynthesis [68]. In this line of reasoning, sugar-sensing mechanisms are likely to play a critical role in regulating plant carbon partitioning and defence responses in mycorrhizal plants upon metal exposure.

## Conclusions

Besides drawing a first picture of shoot proteome modifications upon AM symbiosis and/or heavy metal stress in legume plants, the current work argues for allocation plasticity as the main driving force for Cd extraction in aboveground tissues of *M. truncatula*, a conclusion matching with the view that high biomass producing plants take up a greater metal content than low biomass producing species [29]. Additionally, the retrieved proteomic data also give arguments in favour of the Audet and Charest's hypothesis [32] according to which metal toxicity escape in shoots of mycorrhizotrophic plants is not mediated through an intrinsic tolerance mechanism typical of *A. thaliana*, but is rather supported by the recruitment of antioxidant proteins at the expense of the symbiotic sucrose sink. Actually, the ascorbate-glutathione cycle and molecular chaperones recruited at the the proteome level upon Cd stress alleviation in shoots of mycorrhizal *M. truncatula *plants both belong to the generic cell signature elicited in response to abiotic stressors [69,70]. Notably, they were also suggested to participate in Cd detoxification in the model hyperaccumulator *Thlaspi caerulescens *[71], which led us to investigate whether commonalties may be shared upon heavy metal tolerance between mycorrhizal plants and *T. caerulescens*. Remarkably, out of the shoot proteins *T. caerulescens *whose abundance was modified upon Zn/Cd exposures and/or metal-tolerant accessions were CO_2 _and electron transport-associated proteins, an ascorbate peroxidase, a dehydroascorbate reductase, a PDI, a ß-1,3-glucanase, an adenosine kinase having role in SAM regeneration and nicotianamine synthesis. These point out striking resemblances in the mechanisms candidate to heavy metal escape in those two plant systems, although hyperaccumulation was a term coined for plants able to tolerate and accumulate metals in their aboveground tissues in very high levels, e.g up to 10,000 ppm Cd in the shoot biomass [72], which is actually not the case for *M. truncatula*.

## Methods

### Plant material

For comparative purposes with the previously investigated root responses of *M. truncatula *during cadmium stress alleviation by arbuscular mycorrhiza, all the experiments reported in the current work were performed on the plant material described in [22]. It consisted of four batches of *M. truncatula *plants grown as follows: *M. truncatula *cv jemalong 5 were inoculated or not with the AM fungus *Glomus irregulare *DAOM 181602 (formerly known as *Glomus intraradices *N. C. Schenck & G. S. Smith DAOM 181602; [73]) and half of the plants received a Cd(SO_4_)_2 _solution to obtain a final Cd concentration of 2 mg kg^-1 ^substrate, which corresponds to the limit Cd values established for the European Community (http://www.ademe.fr/partenaires/boues/pages/chap32.htm). Plants were grown for 3 weeks under controlled conditions (16 h photoperiod, 23°C/18°C day/night, 60% relative humidity, 220 μEinstein m^-2^.s^-1 ^photon flux density). Control and *G. irregulare*-inoculated plants either Cd treated or not were watered each day with demineralised water and once a week with a nitrogen-enriched nutrient solution [22]. The four treatments thus encompassed non inoculated plants grown without Cd (C), *G. irregulare*-inoculated plants grown without Cd but inoculated with the AM fungus (Gi), non inoculated but Cd-treated plants (Cd), and *G. irregulare*-inoculated and Cd-treated plants (CdGi), which were available from two independent biological experiments. At time of harvest, shoot and roots were weighted; aerial parts of each treatment were further divided into three samples for cadmium quantification, morphological parameter and pigment content measurements, and protein extraction. Biomass measurements, estimation of mycorrhizal parameters of the root systems and Cd quantification were as described in [22].

### Metrics

The tolerance indices for shoots of mycorrhizal and non-mycorrhizal *M. truncatula *plants were calculated according to [29] as the ratio of biomass (g fresh weight) for plants grown in Cd-spiked substrate to plants grown in Cd-free substrate. Modified from the index of biomass partitioning [32], the contribution of shoots to plant biomass was measured as the ratio of shoot biomass (g fresh weight) to that of whole plant. Root-to-shoot translocation rates for Cd were calculated in mycorrhizal and non-mycorrhizal plants as the ratio of the Cd amount (μg) in shoots to that in root [27,30]. Cd partitioning indices corresponded to the metal quantities (μg) mobilized in plant organs [28].

### Shoot morphological analyses

Shoot branch enumeration and leaf area measurement were performed for two biological experiments on three replicates, each consisting of six plants, after image capture by using the experimental design set up by [74]. Briefly, for plant leaf area measurement, pictures were analysed using the software Visiolog 5.4 (Noesis, Les Ulis, France) in order to estimate the projected leaf area. The latter was determined by comparing pixel value for each plant to pixel value of a standard of known area.

### Chlorophyll content measurement

Pigments were extracted with 80% acetone and absorbance measured at 646 and 665 nm. Chlorophyll *a *(Chl *a*) and chlorophyll *b *(Chl *b*) concentrations were calculated according to standard equations [75]. Chlorophyll content was determined for two biological experiments using three replicates per treatment, each consisting of five plants.

### Chlorophyll fluorescence measurement

Chorophyll *a *fluorescence induction kinetics were measured as previously described [76] on dark adapted (15 min) attached leaves at room temperature. The activity of PSII was evaluated using the JIP-test based on the Chl *a *Polyphasic Fluorecence Transient O-J-I-P [38]. Performance indices based on the JIP-test were calculated as described earlier [77]. The equations used to derive two types of parameters were as follows: (i): The photosynthetic efficiencies at the onset of illumination, *i.e. *the maximum quantum yield of PSII *φ*Po = TR0/ABS = Fv/Fm (where TR and ABS denote the trapped and absorbed excitation energy fluxes); the probability that an electron moves further than Q_A _(*i.e. *electron transfer (ET) *ψ*o = ET0/TR0; the quantum yield for electron transport *φ*Eo = ET0/ABS; the maximum quantum yield of nonphotochemical deexcitation *φ*Do = DI0/ABS, (ii): The vitality indices, *i.e. *the density of RCs per Chl RC/ABS = (RC/TR_0_)(TR_0_/ABS), the conformation term for primary photochemistry *φ*Po/(1-*φ*Po) = TR0/DI_0_, the conformation term for the thermal reactions (nonlight-depending reactions beyond Q_A_^-^) *ψ*o/(1-*ψ*o) = ET0/dQ_A_^-^/dt_0_), the performance indices for energy conservation from photons absorbed by PSII and per CS to the reduction of intersystem electron acceptors PI_ABS _and PI_CS_, respectively and, the performance index for energy conservation from photons absorbed by PSII to the reduction of PSI end acceptors PI_Tot_. The data shown represent an average of 5 to 6 independent measurements per treatment and related statistical data are provided in additional file 2. Rapid response curves of photosynthesis *versus *irradiance were also measured. The quantum yield of PSII photochemistry (Y') was recorded on two different attached leaves for six plant replicates per treatment using 1-sec saturating pulses applied after every 20 sec of illumination with photosynthetically active radiation (PAR) at intensities ranging from 0 to 800 μmol photons m^-2 ^sec^-1^, increased stepwise (standardized automatic recording developed by Walz) [77]. The relative linear electron transport rate (ETR) was calculated by the equation ETR = 0.84 * R * PAR * Y' [78]. It was assumed that 84% of the incident quanta were absorbed (factor 0.84), and that the fraction of the absorbed quanta distributed to PSII (factor R) was 0.6 for wt [79].

### Protein extraction

Proteins were phenol-extracted from shoot tissues (1.5 g) as previously described [80]. Shoot were ground into liquid nitrogen and homogenised in 10 ml of 0.5 M Tris-HCl, pH 7.5, lysis buffer containing 0.7 M sucrose, 50 mM EDTA, 0.1 M KCl, 10 mM thiourea, 2 mM PMSF and 2% (v/v) β-mercaptoethanol. One volume of Tris-buffered phenol was added and, after mixing for 30 min, the phenolic phase was separated by centrifugation and rinsed with another 10 ml of lysis buffer. Proteins were precipitated overnight at -20°C after adding 5 volumes of methanol containing 0.1 M ammonium acetate. The pellet recovered by centrifugation was rinsed with cold methanol and acetone, dried under nitrogen gas and resuspended into 200 μl of 9 M urea, 4% w/v CHAPS, 0.5% v/v Triton X-100, 100 mM DTT and 2% v/v IPG buffer pH 3-10 (Amersham Biosciences). Lipids and nucleic acids were removed by a 30 min ultracentrifugation step at 170,000 *g *(Airfuge, Beckman Coulter). The protein content of the supernatant was quantified by the modified Bradford method as described in Ramagli and Rodriguez [81] using BSA as a standard. In the two independent biological experiments that were performed, proteins were extracted for each treatment from three shoot samples, each consisting of six plants.

### 2-DE analysis

2-DE was performed as described previously [22]. Precast 18 cm nonlinear pH 3-10 IPG strips (Amersham Biosciences) were rehydrated overnight with 600 μg of shoot proteins in 350 μl of 8 M urea, 2% v/v CHAPS, 20 mM DTT, 2% v/v IPG buffer pH 3-10 and bromophenol blue. Isoelectofocusing was carried out for 71 kVh using a gradually increasing voltage at 20°C. Strips were then either stored at -80°C or immediately equilibrated. The second dimension was performed onto homemade 12% pH 8.8 SDS-polyacrylamide gels (Hoefer DALT, Amersham Biosciences). Electrophoresis was run at 10°C for 1 h at 35 V, and then at 80 V until the dye front reached the bottom of the gels. For each treatment, 2-DE was performed for three different shoot protein samples, each consisting of six pooled shoot systems, and two independent biological experiments were analysed. The 2-DE gels were stained with Coomassie Brilliant Blue according to [82].

### Image analysis

Stained gels were scanned using the Odyssey Infrared Imaging System (LI-COR Biosciences, GmbH, Germany) at 700 nm with a resolution of 169 μm. Image analyses were carried out with the Progenesis SameSpots version 2.0 software (nonlinear dynamics) according to manufacturer's instructions. Quantification was performed independently for two biological experiments, corresponding to a total number of 24 gels (4 treatments × 3 independent analytical gels × 2 biological replicates). For each treatment, only protein spots showing significant abundance modification in the two independent biological experiments were considered as differentially accumulated.

### In gel digestion and MALDI-TOF analysis

Following extensive gel washing with water, spots of interest were manually excised with tips, dried and stored at room temperature before mass spectrometry analyses. Gel plugs were washed until de-staining in 100 μl of a 50% acetonitrile/50mM hydrogenocarbonate pH 8 solution and then dried under vacuum. After rehydratation in 10 μl of 50 mM ammonium hydrogenocarbonate pH 8 containing 0.1 μg of porcine trypsin (Promega), samples were incubated overnight (16-18 h) at 37°C. Peptide masses from digested proteins were obtained using a MALDI-TOF-MS equipped with a N_2 _laser (337 nm, 20 Hz, 3ns impulsion) (Voyager DE super STR, Applied Biosystems). Samples were irradiated in a matrix (α-cyano-4-hydroxycinnamic acid 4 mg/ml) and spectra were acquired in reflectron mode within a 700 to 3500 Da mass range and a 130 ns delay extraction time. Internal calibration was performed using trypsin peptide masses within a 500 to 5000 Da range.

PMF search was performed as described in [83] on SwissProt and on the two clustered EST *M. truncatula *database available online (http://medicago.toulouse.inra.fr/Mt/EST/DOC/MtB.html) according to [84]. The first one, named MtC, contained 6350 clusters defined from three root EST libraries (24347 ESTs) of a Genoscope project (http://www.cns.fr/). The clustering process has been previously described in [85]. The second one, named MtD, was obtained using the same process on the *M. truncatula *ESTs (approximately 180000 ESTs) available at the Institute for Genomic Research (http://compbio.dfci.harvard.edu/tgi/). It contained 21400 clusters defined from EST libraries corresponding to different *M. truncatula *tissues. Search for PMF matches was performed in the clustered EST *M. truncatula *databases using the protein prospector software (http://prospector.ucsf.edu/prospector/mshome.htm) and in SwissProt using the profound software (http://prowl.rockefeller.edu/prowl-cgi/profound.exe). For peptide matching, a minimum of four peptides matches and 15% sequence coverage, a maximum of one miscleavage, and peptide modifications by carboxyamidomethylcysteine, methionine sulfoxide, and pyro-glutamic acid or acetylated N-terminal residue, were accepted. The maximum tolerance for peptide mass matching was limited to 20 ppm.

### Data analyses

Means were compared using analysis of variance (ANOVA, *p *< 0.05) using STATISTICA (version 7.1 StatSoft, Inc., 2005, FR; http://www.statsoft.fr). When necessary, data were subjected to arcsin transformation before comparison. For image analysis quantification, homogeneity of the variance was tested and data were subjected to square root transformation when the variances among treatments were not homogeneous. The Tukey's test was used as a *post hoc *test when ANOVA showed significance. The groups of proteins that responded to Cd and/or AM fungal colonisation relative to non-treated plants were further compared using GENESIS clustering (version 1. 7. 2; Graz University of Technology; Institute for Genomics and Bioinformatics). For that purpose, quantitative variations in protein abundance between treatments were represented by Log2 ratios of normalized volume obtained by SameSpots image analysis.

## Authors' contributions

AA participated in experimental design, conducted the bulk of experimental work, performed 2D-electrophoresis and statistical analyses. GR participated in data analysis and drafted the manuscript. FR participated in the experimental work. BS and MB performed chlorophyll fluorescence measurements, help to draft the manuscript and were involved in revising the manuscript critically. CH performed mass spectrometry analyses. VGP, EDG and SAS conceived, designed and supervised the study and were involved in revising the manuscript critically. All authors have read and approved the final manuscript.

## Supplementary Material

Additional file 1 **Impact of cadmium (Cd) and/or *G. irregulare *inoculation (Gi) on biomass (g fresh weight) of 3-wk old *M. truncatula *plants relative to those non-treated (C), as presented in figure **2. For two independent biological experiments (Exp), histograms represent means of three replicates (means ± SD, n = 3). Means marked with different letters indicate significant difference at *p *< 0.05. Click here for file

Additional file 2 ***p *values (n ≥ 5) relative to the parameters of the OJIP-test and relative electron transport rate presented in figure **4. The values below 0.05 are indicated in bold. Click here for file

Additional file 3 **Characteristics of the proteins identified in *Medicago truncatula *shoots, whose accumulation was modified in response to *Glomus irregulare *plant inoculation and/or cadmium supply relative to non-treated plants**. This table includes the annotation, accession number, peptide sequences, percent coverage of the complete sequence, experimental and theoretical Mw and pI of the 23 proteins detected differentially accumulated in this study (n = 3, *p *< 0.05). Click here for file

Additional file 4 **Mass spectrometry data supplied in the mzML format**. Click here for file
