# Supplementary figures and images for: Arbuscular mycorrhizal symbiosis elicits shoot proteome changes that are modified during cadmium stress alleviation in Medicago truncatula
Source: BMC Plant Biol. 2011 May 5;11:75. doi: 10.1186/1471-2229-11-75 (PMC3112074; doi:10.1186/1471-2229-11-75)

## Slide 1
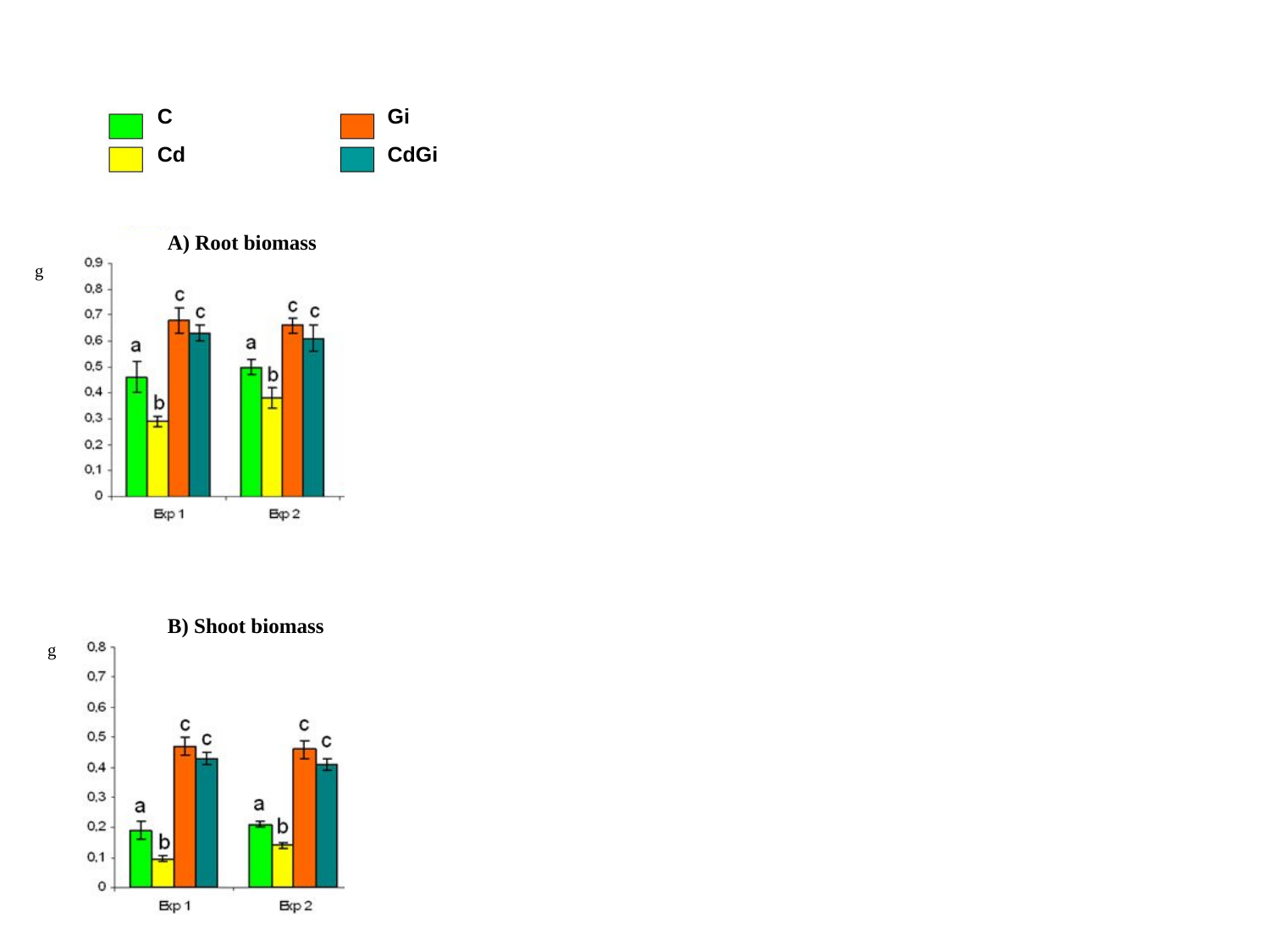

C
Cd
Gi
CdGi
C
g
Racines
Organes aériens
A) Root biomass
B) Shoot biomass
g

Supplement: Additional file 1 — Impact of cadmium (Cd) and/or G. irregulare inoculation (Gi) on biomass (g fresh weight) of 3-wk old M. truncatula plants relative to those non-treated (C), as presented in figure 2. For two independent biological experiments (Exp), histograms represent means of three replicates (means ± SD, n = 3). Means marked with different letters indicate significant difference at p < 0.05. [file 1471-2229-11-75-S1.PPT]
