# Supplementary material for: Arbuscular mycorrhizal symbiosis elicits shoot proteome changes that are modified during cadmium stress alleviation in Medicago truncatula
Source: BMC Plant Biol. 2011 May 5;11:75. doi: 10.1186/1471-2229-11-75 (PMC3112074; doi:10.1186/1471-2229-11-75)
Supplement: Additional file 2 — p values (n ≥ 5) relative to the parameters of the OJIP-test and relative electron transport rate presented in figure 4. The values below 0.05 are indicated in bold. [file 1471-2229-11-75-S2.DOC]

|  | Cd vs C | Gi vs C | CdGi vs C | CdGi vs Cd |
| --- | --- | --- | --- | --- |
| PI(ABS) | 0.11406 | **0.00227** | **0.01969** | **0.00004** |
| PI(Total) | 0.10367 | 0.06707 | 0.16879 | 0.22126 |
| PI(CS) | 0.13633 | **0.00021** | **0.00047** | **0.00005** |
| ψ_0_/(1-ψ_0_) | 0.40719 | **0.01513** | **0.01513** | **0.00012** |
| φP_0_/(1-φP_0_) | **0.0043** | **0.01254** | **0.00222** | **0.00002** |
| RC/ABS | 0.06817 | **0.01623** | 0.29788 | **0.00812** |
| Ψ_0_ | 0.43308 | **0.00245** | **0.01311** | **0.00434** |
| φD_O_ | **0.00487** | **0.01340** | 0.28928 | **0.00019** |
| φE_0_ | 0.28778 | **0.00208** | **0.01184** | **0.00074** |
| F_v_/F_m_ | **0.00487** | **0.01340** | **0.02390** | **0.00019** |
| ETR_MAX_ | **0.02882** | **0.00024** | **2.4E-05** | **0.00048** |
