# Supplementary material for: Arbuscular mycorrhizal symbiosis elicits shoot proteome changes that are modified during cadmium stress alleviation in Medicago truncatula
Source: BMC Plant Biol. 2011 May 5;11:75. doi: 10.1186/1471-2229-11-75 (PMC3112074; doi:10.1186/1471-2229-11-75)
Supplement: Additional file 3 — Characteristics of the proteins identified in Medicago truncatula shoots, whose accumulation was modified in response to Glomus irregulare plant inoculation and/or cadmium supply relative to non-treated plants. This table includes the annotation, accession number, peptide sequences, percent coverage of the complete sequence, experimental and theoretical Mw and pI of the 23 proteins detected differentially accumulated in this study (n = 3, p < 0.05). [file 1471-2229-11-75-S3.DOC]

| **Spot ^a)^** | **Identification ^b)^** | **Accession ^c)^** | **Peptides** | **Cov.^d)^** | **Ex. pI/Mw^e)^** | **Th. pI/Mw^f)^** |
| --- | --- | --- | --- | --- | --- | --- |
| **1** | Photosystem I protein PsaD | P32869 | TEAASVTTK | 39 | 8.8/23 | 9.5/23 |
|  |  |  | AQVEEFYVITWESPK |  |  |  |
|  |  |  | EQIFEMPTGGAAIMR |  |  |  |
|  |  |  | EGPNLLK |  |  |  |
|  |  |  | KEQCLALGNR |  |  |  |
|  |  |  | EQCLALGNR |  |  |  |
|  |  |  | YQFYR |  |  |  |
|  |  |  | VFPNGEVQYLHPK |  |  |  |
|  |  |  | QGVGQNFR |  |  |  |
| **2** | Photosystem I reaction centre subunit IV/PsaE | MtC60681 | YPVVVR | 18 | 8.7/17 | 9.8/15 |
|  |  |  | ESYWYK |  |  |  |
|  |  |  | TRYPVVVR |  |  |  |
|  |  |  | GTGSVVAVDQDPK |  |  |  |
| **3** | Probable protein 2 precursor 0EE2 subunit of oxygne evolving system of PSII | MtC10008 | EFPGQVLR | 30 | 5.3/24 | 7.6/28 |
|  |  |  | QYFNISVLTR |  |  |  |
|  |  |  | HQLITATVNDGK |  |  |  |
|  |  |  | SITDYGSPEEFLSK |  |  |  |
|  |  |  | TNTDFLPYNGDGFK |  |  |  |
|  |  |  | YEDNFDATSNVSVLVQTTDK |  |  |  |
|  |  |  | YEDNFDATSNVSVLVQTTDKK |  |  |  |
| **4**  Additional file 2 continued | Photosystem II polypeptide manganese-stabilizing protein | MtD14660 | VPFLFTIK | 17 | 5.2/34 | 5.8/28 |
|  |  |  | LTFDEIQSK |  |  |  |
|  |  |  | NTPLAFQNTK |  |  |  |
|  |  |  | RLTFDEIQSK |  |  |  |
|  |  |  | GASTGYDNAVALPAGGR |  |  |  |
|  |  |  | DGIDYAAVTVQLPGGER |  |  |  |
|  |  |  | LTYTLDEIEGPFEVSSDGSVK |  |  |  |
|  |  |  | QLVASGKPESFSGEYLVPSYR |  |  |  |
|  |  |  | ITLSVTQTKPETGEVIGVFESIQPSDTDLGAK |  |  |  |
| **5** | RuBisCO small subunit | O65194 | KFETLSYLPPLTEDQLAK | 57 | 6.3/10 | 8.7/20 |
|  |  |  | FETLSYLPPLTEDQLAK |  |  |  |
|  |  |  | EVEYLIR |  |  |  |
|  |  |  | KGWVPCLEFELEK |  |  |  |
|  |  |  | GWVPCLEFELEK |  |  |  |
|  |  |  | GFVYR |  |  |  |
|  |  |  | ENHSSPGYYDGR |  |  |  |
|  |  |  | LPLFGATDSSQVLK |  |  |  |
|  |  |  | AAYPESFIR |  |  |  |
|  |  |  | IIGFDNVR |  |  |  |
|  |  |  | QVQCISFIAHTPATY |  |  |  |
| **6** | Glucan endo-1,3-beta-D-glucosidase | Q9ZP12 | SNGIDKMR | 42 | 5.1/37 | 4.7/36 |
|  |  |  | IYFPDEQALQALK |  |  |  |
|  |  |  | GSNIELILDVAK |  |  |  |
|  |  |  | YVTPYAQDVK |  |  |  |
|  |  |  | YVTPYAQDVKIK |  |  |  |
|  |  |  | VSTAIDMTLIGTSYPPNDGAFTDQAK |  |  |  |
|  |  |  | QYLQPIIDFLK |  |  |  |
|  |  |  | QSISLDYALFK |  |  |  |
|  |  |  | QQGNNDVGYQNLFDAQLDSVYAALEK |  |  |  |
|  |  |  | RPGAIETYLFAMFDENQK |  |  |  |
|  |  |  | TGAATEQHFGLFNPDK |  |  |  |
| **7**  Additional file 2 continued | Phosphoglycerate mutase | MtC00348 | FDNFDR | 23 | 5.5/67 | 5.4/60 |
|  |  |  | SGYFDEK |  |  |  |
|  |  |  | LHILTDGR |  |  |  |
|  |  |  | LDQVQLLLK |  |  |  |
|  |  |  | GIDAQIASGGGR |  |  |  |
|  |  |  | IFEGDGFNYIK |  |  |  |
|  |  |  | HYLVSPPEIDR |  |  |  |
|  |  |  | SVGPIVDGDAVVTFNFR |  |  |  |
|  |  |  | ESFETGTLHLIGLLSDGGVHSR |  |  |  |
|  |  |  | VQILTSHTLEPVPIAIGGPGLTPGVR |  |  |  |
| **8** | Phosphoglycerate mutase | MtC00348 | ALEYEK | 24 | 5.5/67 | 5.4/60 |
|  |  |  | FDNFDR |  |  |  |
|  |  |  | SGYFDEK |  |  |  |
|  |  |  | LHILTDGR |  |  |  |
|  |  |  | QGAPEHWR |  |  |  |
|  |  |  | GIDAQIASGGGR |  |  |  |
|  |  |  | IFEGDGFNYIK |  |  |  |
|  |  |  | HYLVSPPEIDR |  |  |  |
|  |  |  | SVGPIVDGDAVVTFNFR |  |  |  |
|  |  |  | ESFETGTLHLIGLLSDGGVHSR |  |  |  |
|  |  |  | VQILTSHTLEPVPIAIGGPGLTPGVR |  |  |  |
| **9** | Fructose-bisphosphate aldolase cyplasmic isozyme | P46257 | YHDELIANAAYIGTPGK | 28 | 6.4/46 | 6.3/38 |
|  |  |  | RLSSISVENVESNR |  |  |  |
|  |  |  | LSSISVENVESNR |  |  |  |
|  |  |  | GTVELAGTDGETTTQGLDGLGAR |  |  |  |
|  |  |  | YYEAGAR |  |  |  |
|  |  |  | IGPNEPSEHSIHENAYGLAR |  |  |  |
|  |  |  | VAPEVVAEHTVR |  |  |  |
|  |  |  | AAQEALLTR |  |  |  |
| **10**  Additional file 2 continued | Cyclin | MtD27225 | RLPFPGR | 21 | 8.8/34 | 8.8/32. |
|  |  |  | LLLELNR |  |  |  |
|  |  |  | DVLAMSLAPK |  |  |  |
|  |  |  | TRVILDVGCGVASFGGFLFDR |  |  |  |
| **11** | VDAC1.1 | Q6W2J5 | DDLLGALTLNEK | 20 | 8.9/36 | 8.92/29 |
|  |  |  | KGELFVGDVNTQLK |  |  |  |
|  |  |  | FTVSTYSPTGVAITSSGTK |  |  |  |
|  |  |  | VDTESNLFTTITVTEPAPGVK |  |  |  |
| **12** | Chaperonin 21 | Q9M5A8 | MATTQLTASSISTRNLSSFER | 53 | 5.7/25 | 8.5/26 |
|  |  |  | HTTVKPLGDR |  |  |  |
|  |  |  | YAGTEVEFDGSK |  |  |  |
|  |  |  | DEDIVGILETEEVK |  |  |  |
|  |  |  | DLKPLNDR |  |  |  |
|  |  |  | DLKPLNDRVLIK |  |  |  |
|  |  |  | TAGGLLLTEATK |  |  |  |
|  |  |  | TAGGLLLTEATKDKPSIGTVIAVGPGPVDDEGNR |  |  |  |
|  |  |  | DKPSIGTVIAVGPGPVDDEGNR |  |  |  |
|  |  |  | KPLSILPGNTVLYSK |  |  |  |
|  |  |  | YAGNDFK |  |  |  |
|  |  |  | DGSDYIALR |  |  |  |
| **13** | Chaperone DnaK | Q1SKX2 | LVGQIAKR | 28 | 5.2/73 | 5.2/76 |
|  |  |  | QAVVNPENTFFSVK |  |  |  |
|  |  |  | SFAAEEISAQVLR |  |  |  |
|  |  |  | AVVTVPAYFNDSQR |  |  |  |
|  |  |  | IAGLEVLR |  |  |  |
|  |  |  | IINEPTAASLAYGFER |  |  |  |
|  |  |  | QALQRLTETAEK |  |  |  |
|  |  |  | HIETTLTR |  |  |  |
|  |  |  | TPVENSLR |  |  |  |
|  |  |  | DIDEVILVGGSTR |  |  |  |
|  |  |  | IPAVQELVK |  |  |  |
|  |  |  | SEVFSTAADGQTSVEINVLQGER |  |  |  |
| Additional file 2 continued |  |  | LDGIPPAPR |  |  |  |
|  |  |  | FDIDANGILSVAAIDK |  |  |  |
|  |  |  | KQDITITGASTLPGDEVER |  |  |  |
|  |  |  | QDITITGASTLPGDEVER |  |  |  |
|  |  |  | MVNEAER |  |  |  |
| **14** | RNA-binding precursor | MtC10507 | TFNSGLR | 16 | 5.1/34 | 5.2/37 |
|  |  |  | FSGFELDGR |  |  |  |
|  |  |  | VNVAEERPR |  |  |  |
|  |  |  | VESAQVVYDR |  |  |  |
|  |  |  | LAQLFEQSGTVEIAEVIYNR |  |  |  |
| **15** | RuBisCO activase | Q40281 | VYDDEVR | 30 | 5.5/44 | 6.8/47 |
|  |  |  | FYWAPTR |  |  |  |
|  |  |  | NFLTLPNIK |  |  |  |
|  |  |  | SFQAELVFAK |  |  |  |
|  |  |  | YLEGAALGDANQDAIK |  |  |  |
|  |  |  | GLAYDISDDQQDITR |  |  |  |
|  |  |  | IVDTFPGQSIDFFGALR |  |  |  |
|  |  |  | VPIIVTGNDFSTLYAPLIR |  |  |  |
| **16** | RuBisCO activase 1 | Q7X9A0 | EAADIIR | 30 | 5.4/44 | 6.8/47 |
|  |  |  | VYDDEVR |  |  |  |
|  |  |  | FYWAPTR |  |  |  |
|  |  |  | NFLTLPNIK |  |  |  |
|  |  |  | DGPPVFEQPK |  |  |  |
|  |  |  | SFQAELVFAK |  |  |  |
|  |  |  | YLEGAALGDANQDAIK |  |  |  |
|  |  |  | GLAYDISDDQQDITR |  |  |  |
|  |  |  | LLEYGNMLVSEQENVK |  |  |  |
|  |  |  | IVDTFPGQSIDFFGALR |  |  |  |
|  |  |  | VPIIVTGNDFSTLYAPLIR |  |  |  |
| **17**  Additional file 2 continued | RuBisCO small subunit 3A | P07689 | EAADIIR | 26 | 5.3/44 | 6.8/47 |
|  |  |  | VYDDEVR |  |  |  |
|  |  |  | FYWAPTR |  |  |  |
|  |  |  | NFLTLPNIK |  |  |  |
|  |  |  | DGPPVFEQPK |  |  |  |
|  |  |  | SFQAELVFAK |  |  |  |
|  |  |  | YLEGAALGDANQDAIK |  |  |  |
|  |  |  | GLAYDISDDQQDITR |  |  |  |
|  |  |  | LLEYGNMLVSEQENVK |  |  |  |
|  |  |  | IVDTFPGQSIDFFGALR |  |  |  |
|  |  |  | VPIIVTGNDFSTLYAPLIR |  |  |  |
| **18** | DHAR class glutathione transferase | MtC10060 | VHNYIK | 30 | 5.7/24 | 6.3/28 |
|  |  |  | VLLTLEEK |  |  |  |
|  |  |  | ANGPFVAGEK |  |  |  |
|  |  |  | LYHLVVALR |  |  |  |
|  |  |  | VTAVDLSLAPK |  |  |  |
|  |  |  | AAEEYIIAGWAPK |  |  |  |
|  |  |  | DSNDGTEQALLAELNALDEHLK |  |  |  |
| **19** | Protein disulfide isomerase precursor | MtC10403 | LSQYDGGR | 54 | 5.3/61 | 5.0/58 |
|  |  |  | FFNTPNAK |  |  |  |
|  |  |  | VVIVGVFPK |  |  |  |
|  |  |  | DFNVEALEK |  |  |  |
|  |  |  | EADGIVEYLK |  |  |  |
|  |  |  | YHEIAEQYK |  |  |  |
|  |  |  | TGAAHQEVEQPK |  |  |  |
|  |  |  | TKEDIIEFIEK |  |  |  |
|  |  |  | SDYDFGHTLNAK |  |  |  |
|  |  |  | VVVGQTLEDIVFK |  |  |  |
|  |  |  | SEPIPETNNEPVK |  |  |  |
|  |  |  | LFKPFDELSVDSK |  |  |  |
|  |  |  | AASILSTHEPPVVLAK |  |  |  |
|  |  |  | EDQVPLIIIQHNDGK |  |  |  |
| Additional file 2 continued |  |  | VVVGQTLEDIVFKSGK |  |  |  |
|  |  |  | FSGEEYDNFIALAEK |  |  |  |
|  |  |  | EFVLTLDNTNFHDTVK |  |  |  |
|  |  |  | QLAPILDEVAVSFQSDADVVIAK |  |  |  |
|  |  |  | QQGVSFLVGDVESSQGAFQYFGLK |  |  |  |
|  |  |  | LDATANDIPTDTFEVQGYPTLYFR |  |  |  |
|  |  |  | FIEESSIPIVTVFNNEPSNHPFVVK |  |  |  |
| **20** | S-adenosylmethionine synthetase | A4PU48 | TNMVMVFGEITTK | 47 | 5.8/46 | 5.6/43 |
|  |  |  | TIGFISDDVGLDADK |  |  |  |
|  |  |  | VLVNIEQQSPDIAQGVHGHFTK |  |  |  |
|  |  |  | TQVTIEYYNENGAMVPVR |  |  |  |
|  |  |  | VHTVLISTQHDETVSNDQIAADLK |  |  |  |
|  |  |  | EHVIKPVIPEK |  |  |  |
|  |  |  | TIFHLNPSGR |  |  |  |
|  |  |  | FVIGGPHGDAGLTGR |  |  |  |
|  |  |  | SGAYVVR |  |  |  |
|  |  |  | SIVANGLAR |  |  |  |
|  |  |  | ENFDFRPGMITINLDLK |  |  |  |
|  |  |  | TAAYGHFGR |  |  |  |
|  |  |  | TAAYGHFGRDDPDFTWEVVKPLK |  |  |  |
| **21** | 2,4-D inducible glutathione S-transferase | O49235 | TYFGGDK | 42 | 6.1/26 | 6.3/25 |
|  |  |  | SLPDQDK |  |  |  |
|  |  |  | FWADYVDK |  |  |  |
|  |  |  | LLEQELGDK |  |  |  |
|  |  |  | VYGFIVEIR |  |  |  |
|  |  |  | SPLLPSDPYQR |  |  |  |
|  |  |  | GYETFGNINVEK |  |  |  |
|  |  |  | SPLLLQMNPVHK |  |  |  |
|  |  |  | SLPDQDKVYGFIVEIR |  |  |  |
|  |  |  | SICESLIAVQYIDEVWNEK |  |  |  |
| **22**  Additional file 2 continued | Ascorbate peroxidase | Q42459 | SYPTVSADYQK | 53 | 5.7/27 | 5.5/27 |
|  |  |  | LAWHSAGTFDSK |  |  |  |
|  |  |  | TGGPFGTIK |  |  |  |
|  |  |  | HQAELAHGANNGLDIAVR |  |  |  |
|  |  |  | EDKPEPPPEGR |  |  |  |
|  |  |  | EDKPEPPPEGRLPDATK |  |  |  |
|  |  |  | AMGLSDQDIVALSGGHTIGAAHK |  |  |  |
|  |  |  | EGLLQLPSDK |  |  |  |
|  |  |  | EGLLQLPSDKALLSDPVFRPLVEK |  |  |  |
|  |  |  | ALLSDPVFRPLVEK |  |  |  |
|  |  |  | YAADEDAFFADYAEAHQK |  |  |  |
| **23** | Chloroplast precursor | MtC60250 | STVLVTGAGGR | 22 | 5.4/29 | 8.9/35 |
|  |  |  | ALFSQITTR |  |  |  |
|  |  |  | ERPNEYIAR |  |  |  |
|  |  |  | IGAADDVFIGDIR |  |  |  |
|  |  |  | AFDLASKPEGTGSPTK |  |  |  |
|  |  |  | AEQYLADSGIPYTIIR |  |  |  |

^a)^ Spots numbered according to Fig. 4

^b)^Identifications according to database annotations

^c)^Mt accession numbers refer to the clustered EST *M. truncatula* database available online (<http://medicago.toulouse.inra.fr/Mt/EST/DOC/MtB.html>)

^d)^Percent coverage of the complete sequence

^e)^Experimental p*I* and Mw as measured by SameSpots using Precision Plus™ Standards (Bio-Rad)

^f)^Theoretical p*I* and Mw as determined using the Compute pI/Mr tool from ExPASy
